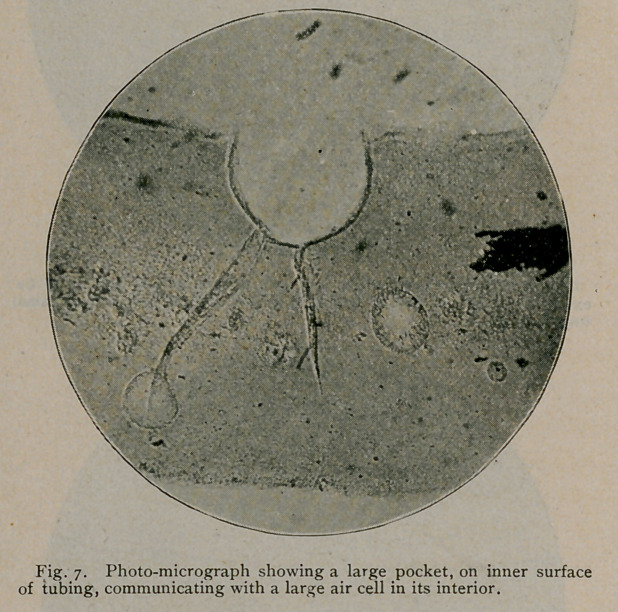# The Great Danger Attending the Use of Long-Tube Nursing Bottles

**Published:** 1900-09

**Authors:** Ernest Wende

**Affiliations:** Buffalo, N. Y.; Health Commissioner, 471 Deleware Avenue


					﻿THE GREAT DANGER ATTENDING THE USE OF
LONG-TUBE NURSING BOTTLES.
(Illustrated. )
By ERNEST WENDE, M. D., Buffalo, N. Y.,
Health Commissioner.
AS MILK is an almost universal food for bacteria, its reception
in a rubber tube, acquired from a coagulum that is normally
cavernous and unstable, invariably presenting surfaces that are rugose
and unpolished, rendering asepsis an impossibility, must necessarily
augment the conditions favorable for the growth and development of
microorganisms, and finally convert it into a nursery which pro-
duces conditions favorable to cholera infantum.
The microscopic examinations of transverse sections, cut from
unemployed rubber tubes, taken from nursing-bottles procured at the
various drug stores of Buffalo, revealed the fact that they were
manufactured out of rubber sheeting cemented together at their
longitudinal margins to complete the tubes, and the seams thus formed
These sections of rubber tubing show the internal surfaces with
its mechanical imperfections, peculiarly adapted to resist cleansing,
permit decomposition and growth of germs, the formation of toxic
matter to contaminate and poison milk as it flows through them.
were found unfailingly imperfect in their construction, showing,
throughout their entire length, elongated pits and’sinuses, which, in
their functions, can only be likened to our modern bacteriologic “breed
ovens.” In-some instances, this state of affairs was further intensi-
fied in that the tubes were constructed of more than one layer,
between which such enormous spaces were frequently developed,
directly connected with the lumen of the tube by the numerous pits
and sinuses referred to, as to more or less involve their entire cir-
cumference. Moreover, the sections likewise demonstrate that the
material used in the manufacture of this tubing is exceedingly porous,
and that the pores vary amazingly in size and shape. Again,
pockets were often seen communicating with the inner surface of the
tube, while, in several specimens, distinct channels were visible con-
necting similar pits or pockets with cavities in the substance of the
rubber. It would, indeed, be difficult to imagine a more efficient
arrangement for the propagation of germs.
By reflected light, under the microscope, the inner surfaces
exhibit a view apparently made up of roughened and scraggy elevations
and depressions with many openings and clefts, extending deeply
into the texture. All the features of the imperfect seams, by this
means, are easily discernible and can be readily traced and studied
throughout their entire length. Therefore, it is obvious that any
foreign material, gaining entrance to this perilous combination of
pits, sinuses and porosites, cannot be removed by any of the ordinary
methods of cleansing, however vigilant and faithful be the mother or
nurse. Yes, it is extremely doubtful whether bacteria, when once
lodged in these incubators, can be displaced or even destroyed by any
known germicide or chemical.
From the bacteriologic examination of an infected rubber tubing,
we were able to recognise a deposit of decomposing material coating
the inner surface, being the thickest in those portions of the lumen
in close proximity to the nipple and glass tubing. This debris was
found, on further investigation, to consist of coagulated casein, with
innumerable bacteria of varied morphology. The qualitative bacterial
examination of the material demonstrated the bacillus acidi lactici to
be the predominating organism. The staphylococcus pyogenes aureus
and ouidium albicans, with three other distinct species, two of which
belonged to the nonchromogenic bacilli, the third being a chromo-
genic micrococcus, were present, and in addition to several species
not yet completely isolated.
Five decigrams of this grumous conglomeration, dissolved in 2
c. c. of sterile water and injected intraperitoneally into each of three
full-grown guinea-pigs, weighing respectively 200, 254 and 261
grams, produced death of the animals in from forty-eight to sixty-one
hours.
471 Deleware Avenue.
				

## Figures and Tables

**Fig. 1. f1:**
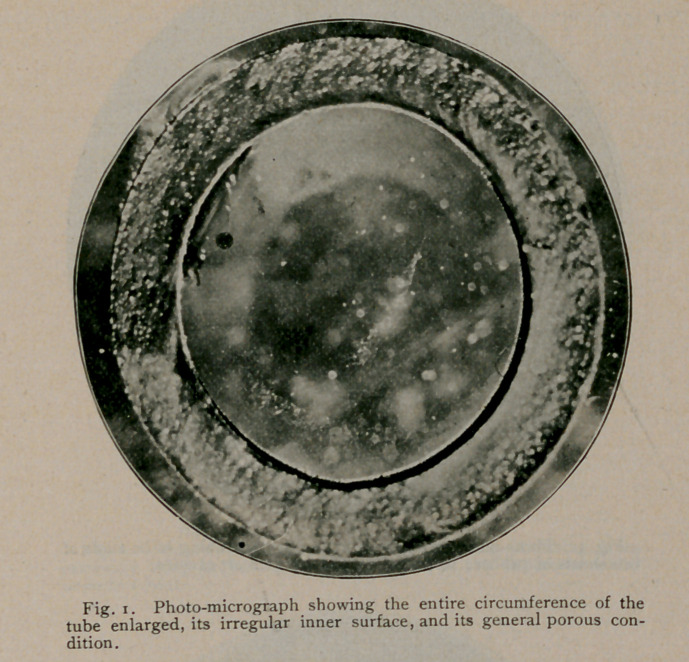


**Fig. 2. f2:**
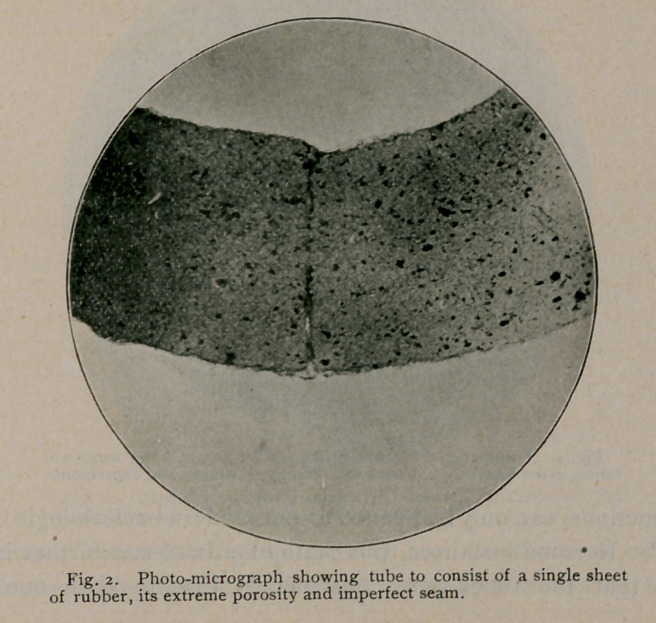


**Fig. 3. f3:**
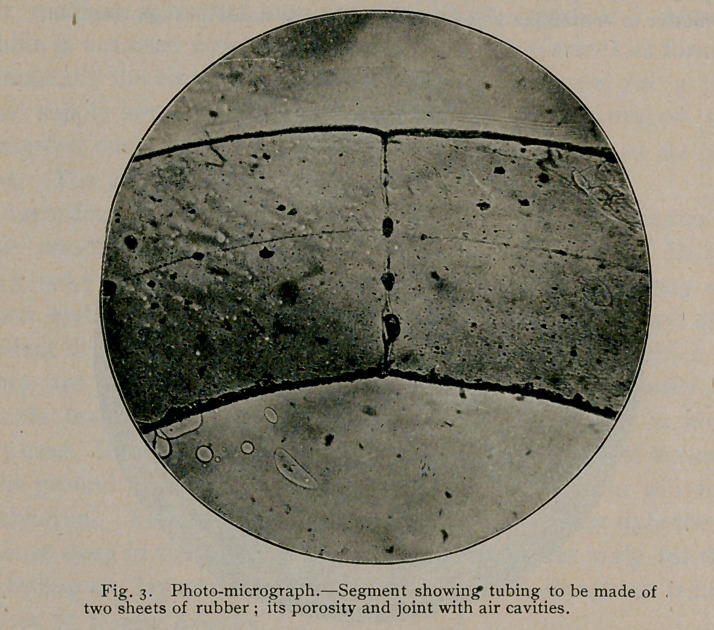


**Fig. 4. f4:**
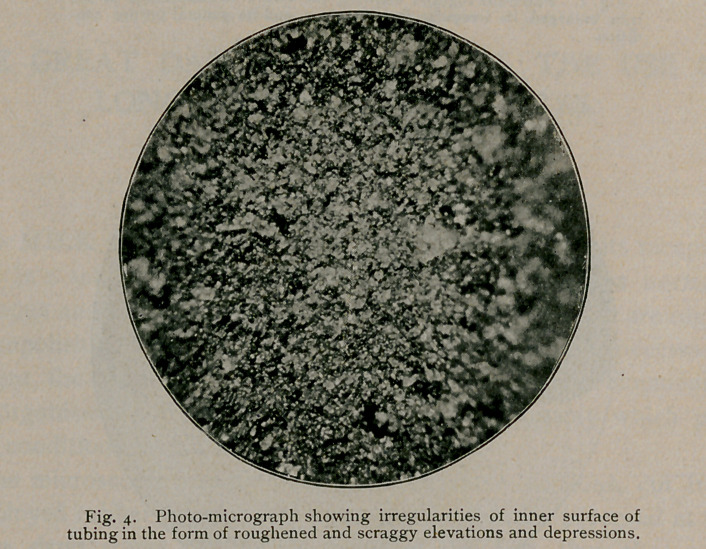


**Fig. 5. f5:**
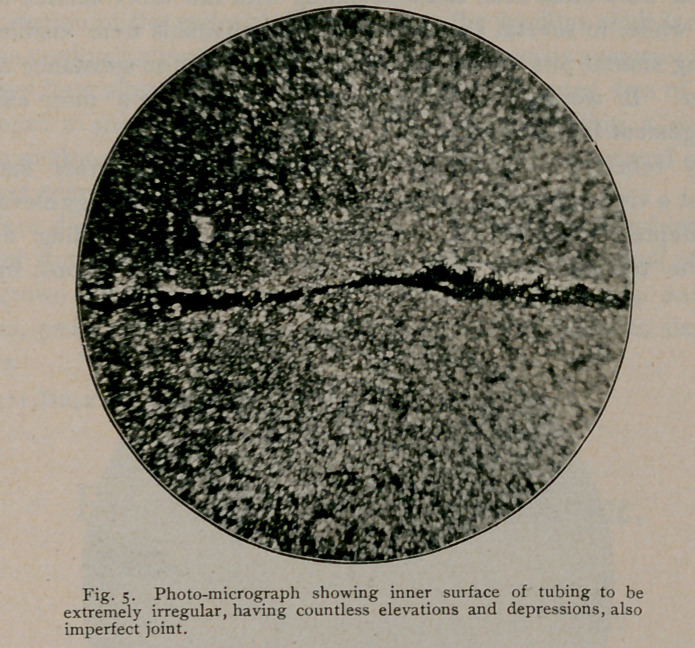


**Fig. 6. f6:**
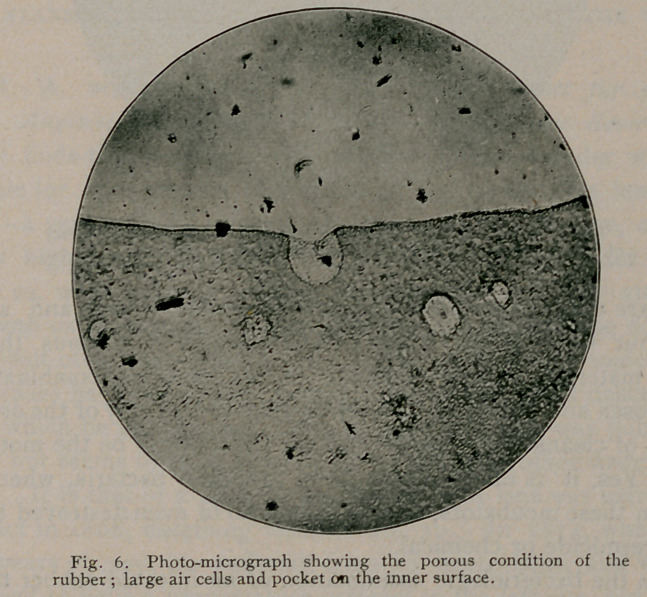


**Fig. 7. f7:**